# Participation in a Swedish cervical cancer screening program among women with psychiatric diagnoses: a population-based cohort study

**DOI:** 10.1186/s12889-019-6626-3

**Published:** 2019-03-18

**Authors:** Erik M. Eriksson, Malena Lau, Claes Jönsson, Chenyang Zhang, Lise-Lotte Risö Bergerlind, Junmei Miao Jonasson, Björn Strander

**Affiliations:** 1 Service Management and Logistics/Centre for Health Care Improvement, Chalmers University of Technology, SE-412 96 Gothenburg, Sweden; 2 Centre for Equity in Healthcare, Gothenburg, Region Västra Götaland Sweden; 3 Department of Healthcare, Gothenburg, Region Västra Götaland Sweden; 4 Sahlgrenska Academy, Institute of Clinical Sciences, Gothenburg, Sweden; 5 Regional Cancer Centre West Sweden, Gothenburg, Sweden; 6 Regional Knowledge Centre for Mental Health, Gothenburg, Region Västra Götaland Sweden; 7 Sahlgrenska Academy, Institute of Medicine, Gothenburg, Sweden

**Keywords:** Cervical cancer screening participation, Pap test, Mental illness, Psychiatric diagnoses, Equity in health

## Abstract

**Background:**

In Sweden, organized screening programs have significantly reduced the incidence of cervical cancer. For cancers overall, however, women with psychiatric diagnoses have lower survival rates than other women. This study explores whether women with psychiatric diagnoses participate in cervical cancer screening programs to a lesser extent than women on average, and whether there are disparities between psychiatric diagnostic groups based on grades of severity.

**Methods:**

Between 2000 and 2010, 65,292 women within screening ages of 23–60 had at least two ICD-10 (International Statistical Classification of Diseases and Related Health Problems – Tenth Revision) codes F20*–F40* registered at visits in primary care or psychiatric care in Region Västra Götaland, Sweden. Participation in the cervical cancer screening program during 2010–2014 was compared with the general female population using logistic regression adjusted for age.

**Results:**

Relative risk for participation (RR) for women diagnosed within psychiatric specialist care RR was 0.94 compared with the general population, adjusted for age. RR for diagnoses outside specialist care was 0.99. RR for psychoses (F20*) was 0.81.

**Conclusions:**

Women with less-severe psychiatric diagnoses participate in the screening program to the same extent as women overall. Women who have received psychiatric specialist care participate to a lesser extent than women overall. The lowest participation rates were found among women diagnosed with psychoses.

## Background

The *World Health Organization* [1] identifies cervical cancer as one of the most serious threats to women’s lives, particularly in low- to middle-income countries. Screening programs offering early detection of precancerous lesions have helped cervical cancer incidence and mortality to decline in developed countries [2]. Sweden has a relatively long history of cervical cancer prevention, having implemented organized screening programs in the mid-1960s [3]. These efforts have significantly reduced the incidence of cervical cancer [4, 5], and the mortality rate has declined by 60% over the last four decades [6].

In Sweden, the standard practice until 2017 has been to invite women between 23 and 60 years of age to take a Papanicolau (Pap) test. The invitation is sent out every three to five years depending on age [7]. In the local context of Region Västra Götaland, over 85% of the women accept the invitation and take a Pap test at a local antenatal clinic [8]. Despite the relative success on an aggregated level, the Swedish cervical cancer screening programs have proven to target segments of the population unequally [9]. Foreign-born women attend to a lower degree than Swedish-born women [10, 11]. It is also suggested that the cervical cancer screening programs fail to attract women living in rural areas [12], women who have sex with women [13], and single women [14]. Moreover, participation rates are lower in socioeconomically disadvantaged areas, suffering from poor educational and income levels as well as high unemployment rates [15, 16]. However, Swedish studies exploring whether mental illness constitute a barrier to attend cervical cancer screening programs are sparse.

Mental illness is suggested to have increased among the Swedish population since the early 1990s, particularly among women between 16 and 34 years of age [17]. Persons with psychiatric diagnoses often have poorer somatic health status and lower life expectancy than the general population [18]. There are also disparities in Swedish cancer care, in which patients with psychiatric diagnoses die from their cancer to a greater extent than other patients [19]. Women with psychiatric diagnoses received their breast cancer diagnosis and treatment at a later stage than other patients with breast cancer, indicating that mentally ill women are not screened to the same extent as other women [19].

International studies have investigated the correlation between psychiatric diagnoses and cervical cancer screening participation. In Canadian [20] and Taiwanese [21] studies, women diagnosed with schizophrenia proved less likely to have a Pap test than women without such a diagnosis. Another Canadian study [22] found that women with psychosis were five times less likely to have a Pap test than women without psychosis. On the contrary, within a group of US women with depression, it could not be found that women with high depressive symptom burden in the subsequent year had lower odds for Pap testing [23]. Naturally, there are variations among the group of mentally ill women. In another Canadian study [24], younger depressed women were found to be more likely to have had a recent Pap test than older depressed women.

Despite the relatively positive results of Swedish cervical cancer prevention, approximately 140 women still die from the disease annually [25], three-quarters of whom had not taken a Pap test within the recommended intervals [26]. Thus, identifying nonparticipants is crucial in order to launch interventions for the relevant group. The present paper addresses the following questions: Compared with invited women overall, to what extent do women with psychiatric diagnoses attend the cervical cancer screening program? Are there differences in participation rates across psychiatric diagnostic groups and grades of severity?

## Methods

### Study design

Despite varieties within the decentralized Swedish healthcare system, the organization of psychiatric care is largely similar across the various regions/councils. As stated in regional guidelines [27], primary care is responsible for early detection and assessment of psychiatric states among the patients seeking care. This level of care is responsible for treating patients with generalized anxiety disorder, panic syndrome, crisis reaction, obsessive compulsive disorder, social phobia, mild to moderate forms of depression, post-traumatic stress syndrome, self-harm, substance abuse, and eating disorders. Besides physicians and nurses, psychologists and psychotherapists work at the primary care centers. Specialist psychiatry is responsible for treating patients who suffer from attention deficit hyperactivity disorder and autism, schizophrenia and other psychoses, bipolar syndrome, relapsing depressions, and severe forms of depression, posttraumatic stress syndrome, self-harm, substance abuse, and eating disorders.

Data was retrieved from three registers. The first is the Vega database, the comprehensive database for healthcare consumption in the Region Västra Götaland. This database includes person-bound diagnosis in all healthcare, in-patient and out-patient, specialized care and primary care. This publicly owned database was established in 2000 and weekly deliveries of data are compulsory from all sectors of healthcare in the region [28]. The second register is the process quality register for cervical screening. This is part of the national quality register for cervical screening [29] and has had 100% coverage since 1993 for cervical smears in the Region Västra Götaland, taken as screening or clinical sample, and in public- and private-run facilities. The third register is the Swedish total population register from the government-run Statistics Sweden [30]. Data was linked by the 12-digit personal number assigned to all citizens in Sweden.

### Cohort definition

As a reference cohort, we used all women registered as residents in the geographical region of Västra Götaland on December 31, 2014 who were aged between 23 and 60. In total, 341,171 women were included in this cohort.

Inclusion criteria for the study cohort included women registered as residents in the region of Västra Götaland on December 312,014; were aged between 23 and 60; who had visited psychiatric outpatient clinics and/or primary care between 2000 and 2010 and, on at least two of these occasions were diagnosed with any of the following diagnosis and ICD-10 (International Statistical Classification of Diseases and Related Health Problems – Tenth Revision) codes: psychoses (F20*–29*); affective disorders (F30*–39*); or phobia, anxiety, stress, etc. (F40*–48*). A woman did not need to have the same diagnosis on both occasions in order to be included in the study cohort. In the Vega database of Region Västra Götaland, at total of 65,292 women were identified in accordance with the above.

Each woman in the study cohort was sorted into a diagnostic group, based on her most severe diagnosis. Consequently, diagnoses within F30 and F40^1^ were graded either S (severe, contact with psychiatric specialist) or L (less severe, no contact with psychiatric specialist). No grading was made of F20, as all women with diagnosis of psychosis had contact with psychiatric specialist. Thus, the diagnoses, ranked from most to least severe, were: (1) F20, (2) F30S, (3) F40S, (4) F30 L, and (5) F40 L.

### Outcome

Participation in the cervical cancer screening program from January 1, 2010 to June 30, 2015 for the study cohort were compared with reference cohort’s participation during the same period of time. Because of the standard practice in which women under 50 years of age are invited to the screening program every three years, and women over 50 years of age are invited every five years, all invited women should have received at least one invitation during the selected period of time. Due to invitation procedures that allow some variance, a five-and-a-half-year outcome period was set.

### Statistical analysis

The analysis was conducted using logistic regression adjusted for age and censored for outcome before exposure. The latter was relevant due to an overlap in the intervals of 2010, meaning there was a risk that women in the study cohort could have participated in the screening program before being exposed for psychiatric diagnoses. Consequently, “censored for outcome before exposure” indicates that “participation” in the screening program for women in the study cohort is relevant only if they had participated after two registered psychiatric diagnoses. However, there were no censored patients in this study. The relative risk (RR) and 95% confidence interval (CI) were calculated. All statistical analysis were performed using R.

## Results

Table 1 describes the baseline characteristics of our study cohort by age and most severe psychiatric diagnosis.

**Table 1:** Baseline characteristics of the study cohort

Baseline characteristics	No. of Women
Age (years)	
23–30	11,288
31–40	19,335
41–50	24,176
51–60	10,493
Diagnosis	
Psychosis F20*–29*	2364
Affective disorder F30*–39*, specialist care	15,858
Phobia, anxiety, stress, etc. F40*–48*, specialist care	122
Affective disorder F30*–39*, not specialist care	27,974
Phobia, anxiety, stress, etc. F40*–48*, not specialist care	18,974
Total	65,292

*All subgroups included

The first row in Table 2 shows the number of visits with one or several psychiatric diagnoses for the study cohort on an annual level. Because the patients may revisit healthcare providers year after year and the cohort gradually increases, these numbers appear to have a cumulative nature. The second row show the number of unique patients calculated on the basis of the first visit with psychiatric diagnosis during the study period.

**Table 2:** Number of visits with psychiatric disease diagnoses and number of unique new patients in the study by year

Year	2000	2001	2002	2003	2004	2005	2006	2007	2008	2009	2010
No. of visits	5300	19,991	37,816	55,258	64,940	69,757	89,995	103,786	108,276	152,644	185,008
Patient’s first time diagnosed	2749	7702	7235	7221	7530	6172	6080	5125	4702	5496	5280

As shown in Table 3, the relative risk for participation (RR) among women within the group that had contact with specialist psychiatric care (FxxS) was 0.94 (*p* < 0.05). The RR for diagnoses without contact with psychiatric specialist care was 0.99 (*p* < 0.05). The RR for women with psychotic diagnoses (F20*–F29*) was 0.81 (*p* < 0.05).

**Table 3:** Participation rates and the relative risks (RR) for participation by group of psychiatric ICD diagnosis and severity as level of care. (S) = Specialist care, (L) = Non-specialist care. Adjustment made for age

Group	Raw Rate	Adjusted Rate	Adjusted Rate L	Adjusted Rate U	Adjusted RR	Adjusted RRL	Adjusted RRU
Reference cohort	88.5	88.5			1.00		
Study cohort	86.0	86.4	86.1	86.7	0.98	0.97	0.98
FxxS (all severe)	82.6	83.1	82.6	83.7	0.94	0.93	0.95
FxxL (all less severe	87.3	87.7	87.4	88.0	0.99	0.99	1.00
F20*–F29* (all)	69.8	71.2	69.4	73.0	0.81	0.78	0.83
F30*–F39* (all)	85.9	86.4	86.0	86.7	0.98	0.97	0.98
F40*–F48* (all)	88.1	88.5	88.0	89.0	1.00	1.00	1.01
F30*–F39* (S)	84.5	85.0	84.4	85.5	0.96	0.95	0.97
F30*–F39* (L)	86.7	87.1	86.7	87.5	0.98	0.98	0.99
F40*–F48* (S)	76.2	76.3	68.2	83.3	0.86	0.77	0.94
F40*–F48* (L)	88.2	88.5	88.1	89.0	1.00	1.00	1.01

*All subgroups included

## Discussion

Despite the effectiveness in reducing cancer mortality [31, 32], participation rates among groups of women in cervical cancer screening programs vary [33]. This study adds to previous knowledge of participation in cervical cancer screening among women with psychiatric diagnoses by highlighting the potential difference in participation among women with severe and less severe such diagnoses. The strengths of the study include a population-based cohort design with a large sample size. Using high-quality registers instead of self-reported exposure and outcome information further adds to its strengths.

This study could not report any considerable differences in risk of participation in the local screening program between the general reference group and the study group with psychiatric illness overall. Due to the large size of the study, almost all differences are statistically significant, but the clinical importance of the difference is small. Similarly, previous research [23] has not found that the odds of taking a Pap test are lower for women with high depressive symptom burden. However, within the study cohort there were important variations in this study. For example, women who attended psychiatric specialist care were less likely to participate in the screening program than women who had received their psychiatric diagnoses in primary care or elsewhere; this most probably reflects differences in the severity of the disease. Most notably, and similar to previous findings [20–22], women with psychosis and obsessive/compulsive disorders in specialist care were least likely to have a Pap test, while women with even severe affective disorders participated in screening to the same extent as the general population.

We constructed a hierarchy of groups of psychiatric diagnoses, which served as the base for selecting the most severe diagnosis when women had more than one. This scale is not validated. Another limitation is that the completeness of the large Vega database is not validated, which means that data from its development phase – the first years after 2000 – could be missing. This bias could theoretically underestimate the true difference. However, this bias seems to be very limited, given that the largest number of unique patient entries was registered in 2001, the second year after the start of the database (Table 3).

We have not had access to other data that could have an association with the outcome, such as socioeconomic deprivation and marital status. Adjusting for such factors could be of further epidemiologic interest, although it is beyond the scope of this study as we aim to study actual participation in the screening program and identify differences across psychiatric diagnosis, not to investigate causality.

The strengths of this study are that it covers the entire population and that outcome data comes from a comprehensive database with total coverage of the population. The criterion that women had to have been diagnosed on at least two occasions in order to be included in the study cohort also provides a validation of psychiatric illness.

Official national reports [19] and previous international studies [34] report higher cancer mortality rates in people with psychiatric diagnoses compared to the population overall. Concerning cervical cancer, some research [35] has not found any differences in the risk of developing cervical cancer between patients with schizophrenia and patients without the diagnosis. Indeed, a Danish study [36] suggested that patients with schizophrenia had a decreased risk of developing cervical cancer compared to other women. Our study indicates that women with psychosis have a 20% lower screening participation than the general population, and thus a lower protection against cervical cancer. If this finding was also valid for breast cancer screening, it could explain part of the pronounced worsened stage-distribution found for patients with psychosis [19].

For women with schizophrenia, it is suggested that good continuity of care increases the likelihood of a Pap test being taken [20]. A general barrier for participation in cervical screening programs is suggested to be its impersonal and anonymous nature [37]. To overcome this, it is considered important not only to focus on printed material and invitations, but also to spread information orally [38] or by films [39], to include women’s social networks in dissemination of information [10, 40, 41], or to arrange special events [42]. Representatives of non-participants may also be invited to identify barriers, propose solutions, and to execute these solutions to their peers [15, 16]. In the local context of this study, the healthcare provider’s suggested actions to overcome inequities include annual counseling for persons with psychiatric illnesses about somatic status, including whether they had participated in mammography and had a Pap test taken [43].

Moreover, it is suggested that participation of persons with serious mental illnesses may vary between different programs, with higher participation in cervical cancer screening than breast, prostate, and colorectal cancers [44]. This suggests that non-participation may be even greater in other screening programs than the cervical cancer screening program of this particular study.

In the local context of Region Västra Götaland in Sweden, the last two decades have seen great efforts to methodically improve knowledge of cervical cancer and increase participation in the local screening program. Consequently, overall participation in the regional cervical cancer screening program is higher than in many other regions in Sweden [29]. These efforts may have benefitted women with psychiatric diagnoses as well, especially explaining the almost identical participation between women with less severe (L) psychiatric diagnoses and women overall in this study.

## Conclusions

Most women in Sweden participate in the cervical screening program. This study has found that women with less severe psychiatric diagnoses participate in the screening program to a similar extent as women overall. While women with severe affective disorders also have a high participation rate, women with other psychiatric diagnoses requiring specialist care, such as psychosis, participate less. This implies that psychiatric specialist care should better support their female patients to participate in the cervical screening programs, and other actors in the healthcare system should better support engagement for the group in the screening program.

## Abbreviations

CI: Confidence interval
ICD-10: International statistical classification of diseases and related health problems – tenth revision
L: Less severe
No.: Number
Pap: Papanicolau
RR: Relative risk
S: Severe
US: United States of America

## Footnotes

1 *All subgroups included.
